# Analysis of Learning Behavior of Human Posture Recognition in Maker Education

**DOI:** 10.3389/fpsyg.2022.868487

**Published:** 2022-05-30

**Authors:** Yueh-Min Huang, An-Yen Cheng, Ting-Ting Wu

**Affiliations:** ^1^Department of Engineering Science, College of Engineering, National Cheng Kung University, Tainan, Taiwan; ^2^Graduate School of Technological and Vocational Education, National Yunlin University of Science and Technology, Yunlin, Taiwan

**Keywords:** human posture recognition, convolutional neural networks, artificial intelligence, learning behavior, maker education

## Abstract

Maker education mainly involves “hands-on” as the core concept and combines various educational theories to redefine interactions between learners and teachers in a learning environment. Identification of meaningful “hands-on” behaviors is crucial to evaluate students’ learning performance, although an instructor’s observation of every student is not feasible. However, such observation is possible with the aid of the artificial intelligence (AI) image processing technique; the AI learning behavior recognition system can serve as the second eyes of teachers, thus accounting for individual differences. However, in previous studies, learning behavior recognition was applied to the traditional or static classroom. A behavior recognition system for identifying “hands-on” actions in the learning context has still not been developed. Therefore, this study designed a human posture evaluation system, obtained human articulation node information from learning field images, and built a learning behavior recognition model suitable for maker education based on the AI convolutional neural network (CNN). A learning behavior model was defined, along with a number of student behavior indexes. Subsequently, the effectiveness of the model and behavior indexes was verified through practical learning activities. The model evaluation results indicated that the proposed model achieved a training accuracy of 0.99 and a model accuracy of 0.83. Thus, the model can be applied to dynamic maker activity learning environments.

## Introduction

Maker education, a teaching model with hands-on as the core philosophy (Blikstein, 2013; Halverson and Sheridan, 2014; Lemieux, 2021), combines various educational theories and integrates science, technology, engineering, and mathematics (Bevan et al., 2015; Martin, 2015; Hsu et al., 2017; Kim et al., 2022). Maker education fosters problem-solving skills and innovative thinking through the application of a learner-centered approach, during which teachers pay more attention to students and promptly assist them in achieving favorable outcomes (Blikstein, 2013; Honey and Kanter, 2013; Halverson and Sheridan, 2014; Rodriguez et al., 2021). However, because of the nonavailability of an adequate number of teachers, providing sufficient attention and appropriate instructions to each student is difficult, particularly in Taiwan, where maker education places a considerable burden on educators because of the higher student–teacher ratio compared with that in Europe and North America.

With advances in technology, artificial intelligence (AI) has emerged in recent years. If an AI-based learning behavior recognition system becomes the second pair of eyes that pays attention to students, teachers may no longer feel exhaustion. In response to the demand for learning behavior recognition, previous studies have built models that recognize student behaviors. On the basis of the frameworks of the convolutional neural network (CNN; Sun et al., 2018; Liu et al., 2019) and region-based fully convolutional network (Li et al., 2019), these studies have obtained human node data using wearable sensors, such as pressure sensors (Xu et al., 2013), three-axis accelerators (Ma et al., 2016), or general imaging and special lenses equipment and technologies such as Kinect (Ashwin and Guddeti, 2019; Chin et al., 2019) and OpenPose (Sun et al., 2018; Liu et al., 2019).

Although previous studies have discussed the applications and advantages of the learning behavior recognition system, most studies have evaluated the system’s educational applications in traditional, static classrooms. Moreover, no model for dynamic classrooms in maker education has been developed. Therefore, this study obtained human node data from the images of actual maker teaching scenes by using a human pose evaluation system to build a model based on the CNN that is applicable to learning behavior recognition in maker education. In addition, this study determined whether a model trained using human node data can be applicable to behavior recognition in a maker education site by testing the images of actual maker teaching scenes.

## Materials and Methods

### Model Building

This study built a model by using the existing system reported by Chen et al. (2020). The modeling process is as follows: first, the operator categorized behavior videos as training data, which were converted into images in advance, and one image was extracted for every two frames. Among the categorized images, 70% were used as the training dataset and 30% as the testing dataset. Subsequently, the system extracted human node data from the training dataset by using the TF-pose-evaluation tool. Second, the operator provided the system with the labeling data of the frame where videos corresponded to behaviors, with the labeling format such as Listen 1 22, indicating that characters from the 1st to 22nd frames denoted the learning behavior. After combining the node data and labeling data, the system calculated the feature data (e.g., the movement speed of the limbs and nodes) of various actions. Subsequently, the features were extracted using the principal component analysis algorithm to reduce the dimension. Finally, the extracted feature data were used to train and build the model by using the DNN classifier with three layers and 50 nodes in each layer. The confusion matrix is a method used to evaluate model performance (Townsend, 1971). The precision and recall of behaviors can be calculated from the values of the confusion matrix, whereas the F1 score considers precision and recall at the same time (Sokolova and Lapalme, 2009; Wiki, 2020). The formula is as follows:


$$Precisionof\;\mathrm{HandsOn}=\frac{A}{A+E+I+M}$$

$$Recallof\;\mathrm{HandsOn}=\frac{A}{A+B+C+D}$$

$$F1\;Scoreof\;\mathrm{HandsOn}=\frac{2}{\frac{1}{Precisionof\;\mathrm{HandsOn}}+\frac{1}{Recallof\;\mathrm{HandsOn}}}$$

Supportof HandsOn=A+B+C+D

$$macro\;avg\;of\;F1\;Score=\frac{F1\_H+F1\_L+F1\_I+F1\_O}{4}$$

$$weighted\;avg\;of\;F1\;Score=\frac{\begin{array}{l} F1\_H\times S\_H+F1\_L\times S\_L+\\ F1\_I\times S\_I+F1\_O\times S\_O \end{array}}{S\_H+S\_L+S\_I+S\_O}$$

### Recording and Analysis of Films

Once the model was established, video analysis was performed. This study adopted the following process to analyze the recorded videos: First, the operator imported a video of the teaching scene into the system and used the TF-pose tool to capture the human node data frame at a frame rate of 20 fps. Then, student behaviors were analyzed using the established model to generate behavioral interpretation videos and data. Finally, the operator summarized the behavioral interpretation data and produced the behavioral interpretation report by using the visualization tool.

### Behavioral Indicators

Maker activities used in this study were related to “fun for gears,” which includes four subactivities. Activity 1: Leading students to observe objects or tools that use gears in life and think about the purpose of using gears. Activity 2: Asking students to observe and measure the gear entity and assemble the gear set to observe the actual rotation phenomenon. Activity 3: Explaining the operation principle of the correction belt and providing the correction belt image to guide students to observe and think. Through group discussions, designing and drawing the correction belt style, determining the movement state, and practicing the usage method required one to three gears. Activity 4: Provide students with wooden gear box parts prepared using laser cutting and provide instructions to guide students to complete the work according to the steps.

Maker activities include diverse content, and their primary purposes are crafting, observing others’ works, and developing diverse ideas from the community; these activities focus on solving various problems (Dougherty, 2012). Crafting, observing, and solving problems are the primary activities in maker education. Therefore, using behavioral indicators as recognition categories instead of directly defining actions is more appropriate for analyzing maker education scenarios. By referring to the definitions of hands-on activities described by Hsiao et al. (2014), Chen et al. (2020), and Zheng et al. (2020) as well as the problem-solving stage and positive learning behaviors, this study designed four types of behavioral indicators. The definitions of off-task actions reported by Taber et al. (1999), Rathvon (1990), and Godwin et al. (2016) were used as the reference for defining activities. The four types of actions were interact (stating and discussing, with apparent gestures or instructing poses), hands-on (writing or building a maker work), listen (standing or sitting, with head-up but no hand movement), and other (playing with learning materials or engaging in actions unrelated to the course).

## Results

### Behavioral Recognition Model

This experiment used video recording tools to capture the images of students’ learning process in groups. A total of six groups, with approximately 3 h of teaching scene videos recorded per group, were included. After the exclusion of the footage of students sitting in, leaving, and class break, Activities 1 and 2 and Activities 3 and 4 were used as Session 1 (approximately 1 h) and Session 2 (approximately 1 h and 40 min), respectively, producing two videos for each group.

Considering the shooting angles and student status, the videos of Groups 2–6 were chosen as the training dataset, and those of Groups 1 and 4 were used as the testing dataset. According to the definitions of the four types of learning behaviors, the related videos were selected from those of Groups 2, 3, 5, and 6 for editing. After editing, each behavioral type contained 80–100 groups of videos, which were converted into images, generating 3,331 interacting actions, 8,120 hands-on actions, 5,826 listening actions, and 3,098 other actions. Because of differences in the duration of behaviors, hands-on activities lasted longer, whereas interactions (i.e., gestures) were shorter; the number of images varied after conversion. Table 1 presents the results of the model obtained using the aforementioned formula and the values of the confusion matrix regarding the model’s forecast and actual values. Model accuracy was determined to be 0.83.

**Table 1:** Model accuracy report.

Accuracy on training set: 0.99				
Accuracy on testing set: 0.83				
Accuracy report:				
	Precision	Recall	F1-score	Support
Interact	0.72	0.72	0.72	630
HandsOn	0.87	0.85	0.86	1,529
Listen	0.89	0.91	0.90	1,223
Other	0.74	0.74	0.74	585

Accuracy			0.83	3,967
Macro avg	0.80	0.80	0.80	3,967
Weighted avg	0.83	0.83	0.83	3,967

**Figure 1:**
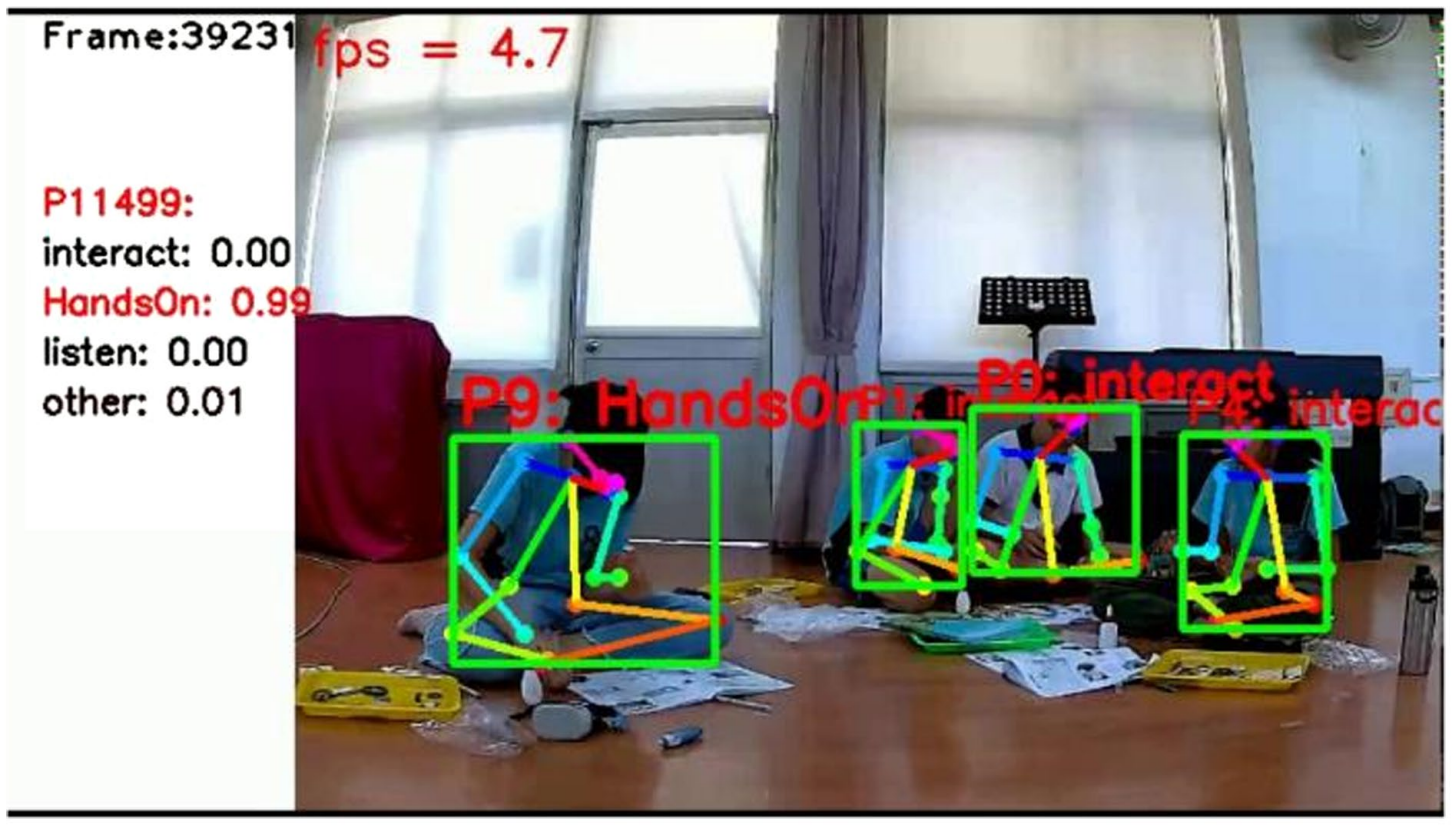
Video screen of behavior recognition.

The video screen of behavioral recognition results is shown in Figure 1, with four students, each surrounded by a tray for placing gears and parts. One student was performing hands-on activities, whereas the other three were sitting together and interacting. The frame value was 39,231, representing the scene between 32 and 33 min after Session 2 began (Activity 4 was started for approximately 3 min); P11499 refers to the number of people selected by the system starting from the analysis to that point in time. Because the human pose evaluation technology did not recognize personal information, the selecting code did not represent the corresponding student. The Interact, Handson, Listen, and Other below P11499 referred to behavioral types in the selection. As shown on the screen, the system indicated that P11499 represented a 99% probability of students’ engaging in hands-on activities; thus, the behavioral type was highlighted in red.

### Visualized Report

**Figure 2:**
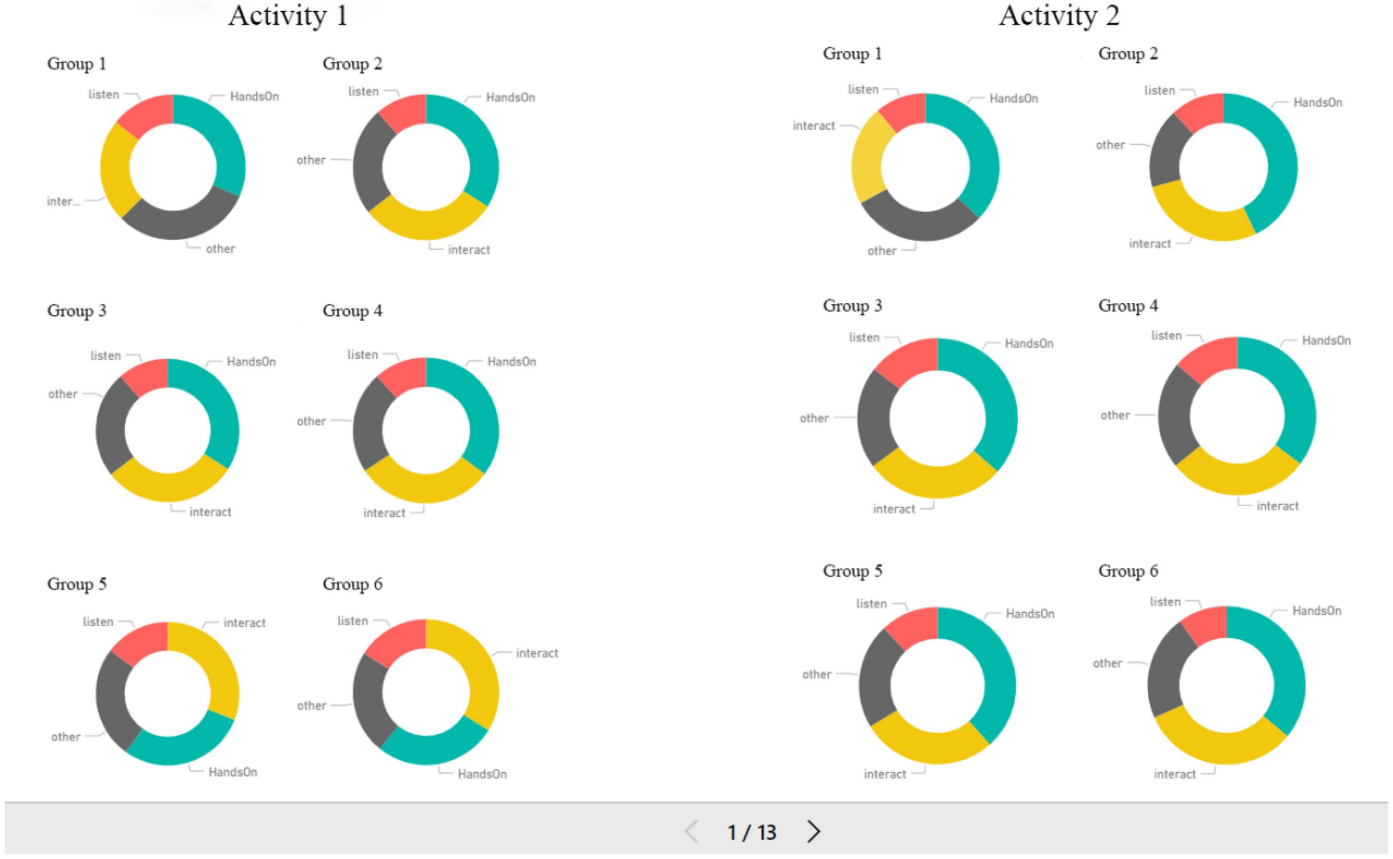
Visualized report of each group’s behaviors.

This study used the system to analyze the videos of teaching scenes, which read and generated data frame by frame. For each group, approximately 70,000 pieces of data were produced in Session 1 and 110,000 in Session 2. After the exclusion of data without judgment, the number of behavior counts was calculated per second per system for each person, and one occurrence in 20 frames was counted as one time (e.g., the system judged three students of a specific group in a specific second, indicating that of the 20 frames, Person A had three frames of hands-on activities and 15 frames of interacting, Person B had 12 frames of hands-on activities and five frames of listening, and Person C had six frames of other behaviors and 10 frames of hands-on activities). Thus, the number of the behavior counts of that group in that second was three hands-on, one listening, one interacting, and one other behaviors; subsequently, summary data were generated according to the total number in 1 min. Furthermore, summary data were imported into visualization software to produce the visualized report released on the Internet (Report link: https://reurl.cc/b5KNpr). The teachers could observe learning behavior counts in the corresponding course time on each group’s visualized page. Each group’s behavior counts are shown in Figure 2.

## Discussion

This study established a model that can be used to recognize student behaviors in maker education. This study generated a visualized report that assisted teachers in understanding student learning behaviors by designing a set of indicators and presentations that met the demand for teaching evaluation. This study speculated that the recognition model and visualized report would support teachers in paying attention to student behaviors in maker activities. The recognition model established in this study had an accuracy of 83%. Despite occasional errors in recognition, the comparison between the on-site observation and report results indicated that the report reflected students’ learning behaviors. Thus, the system for maker learning behavior recognition developed in this study can replace the manual coding of the traditional observation method and can aid in the evaluation of dynamic activity learning environments by teachers and researchers. Moreover, when the number of maker teachers is insufficient, the system and visualized report can serve as the teacher’s second pair of eyes, thus helping educators gain comprehensive knowledge on students’ status and providing appropriate assistance. This study will continuously collect behavioral samples and verify the model’s recognition status to improve the model’s accuracy and applicability.

## Data Availability Statement

The raw data supporting the conclusions of this article will be made available by the authors, without undue reservation.

## Ethics Statement

Written informed consent was obtained from the minor(s)’ legal guardian/next of kin for the publication of any potentially identifiable images or data included in this article.

## Author Contributions

Y-MH, A-YC, and T-TW contributed to conception and design of the study. A-YC organized and analyzed the database. T-TW wrote the draft of the manuscript. Y-MH reviewed sections of the manuscript. All authors contributed to the article and approved the submitted version.

## Funding

This research is partially supported by the Ministry of Science and Technology, Taiwan, R.O.C. under Grant No. MOST 108-2628-H-224-001-MY3, MOST 110-2511-H-224-003-MY3, and MOST 110-2622-H-224-001-CC2.

## Conflict of Interest

The authors declare that the research was conducted in the absence of any commercial or financial relationships that could be construed as a potential conflict of interest.

## Publisher’s Note

All claims expressed in this article are solely those of the authors and do not necessarily represent those of their affiliated organizations, or those of the publisher, the editors and the reviewers. Any product that may be evaluated in this article, or claim that may be made by its manufacturer, is not guaranteed or endorsed by the publisher.
